# Instability in longitudinal childhood IQ scores of Guatemalan high SES individuals born between 1941-1953

**DOI:** 10.1371/journal.pone.0215828

**Published:** 2019-04-25

**Authors:** Liina Mansukoski, Eef Hogervorst, Luis Fúrlan, J. Andres Galvez-Sobral, Katherine Brooke-Wavell, Barry Bogin

**Affiliations:** 1 School of Sport, Exercise and Health Sciences, Loughborough University, Loughborough, United Kingdom; 2 Centro de Estudios en Informática Aplicada, Universidad del Valle de Guatemala, Guatemala City, Guatemala; 3 Centro de Investigaciones Educativas, Universidad del Valle de Guatemala, Guatemala City, Guatemala; University of Zaragoza, SPAIN

## Abstract

Childhood IQ has been used to predict later life outcomes across disciplines in epidemiology, education, and psychology. Most often only a single childhood IQ test is available or is used for these purposes in the belief that IQ is stable across the life course. The primary aim of this study was to examine the longitudinal stability of individuals’ IQ test scores derived from school-age tests. The secondary aim was to investigate the association of the pre-adult scores with later life intelligence scores. The longitudinal pre-adult IQ scores of 42 high socioeconomic status Guatemalans born 1941–1953 were analysed and showed low stability of longitudinal test scores. Fluctuations of >1SD were found for 59.5% of the sample. The same participants, aged 64–76 years, were re-assessed and average pre-adult IQ explained 12% of variance in the older age intelligence score. The reasons behind the longitudinal instability in test scores reported in this study remains unknown but the results suggest single point measurements of intelligence before adulthood should be regarded with some caution.

## Introduction

Previous studies have associated childhood general mental ability, often measured as Intelligence Quotient (IQ) scores, with various later life health outcomes, including risk of dementia and mortality[1–7]. Studies connecting childhood or adolescent mental ability/IQ to later life outcomes assume that there is relative stability in cognitive performance test scores over the developmental period, as often only a single, un-replicated IQ measurement is available or selected to represent early life general mental ability [3,6,8,9]. A systematic review identified a total of nine studies where IQ scores from childhood or early adulthood were investigated longitudinally in relation to later life mortality[1]. None of the studies had repeated childhood IQ measurements; instead, tests were administered only at a single time point, commonly around 11 or 12 years of age [1]. Much literature shows evidence for longitudinal stability of IQ across the life course [10,11], and individuals appear to follow their own ‘tracks’ in terms of broad cognitive ability and are thought to maintain their position relative to others in IQ during the age-related decline–individuals who scored highly compared to peers early in life seem to do so later on as well [12]. Other work has investigated stability of ‘absolute’ IQ scores–that is, repeated tests taken by an individual and reported high levels of fluctuation in scores, particularly during pre-adult years [13–16]. Possible reasons for instability are adverse life events, larger than expected deviations of individual developmental level at the time of the testing and differences between the testing instruments [13–16]. Due to these fluctuations it is important to investigate whether a single timepoint measure of IQ in pre-adult years can be considered representative of childhood mental ability, and how this relates to old age scores.

It has been argued that cognitive ability has two distinct components: fluid intelligence, which measures the influence of biological factors on intellectual development (i.e., heredity, brain injury), and crystallised intelligence which is a manifestation of influence from education, experience, and acculturation [12]. IQ tests are thought to capture both, and remain a widely used measure in life course and epidemiological research [17]. Therefore, it is important to explore the variation seen within and between individuals’ IQ test scores to understand test precision, whether this is related to the type of test used, the administration process, or other less easily identified sources of variability. Finally, in ageing samples, tests measuring aspects of cognitive ability that are thought to be resistant to age-related decline (e.g. word pronunciation) are used to estimate a baseline cognitive performance level, which acts as a proxy for premorbid cognitive ability [18]. The results of cognitive impairment tests can then be adjusted for the estimated premorbid ability [19]. Therefore, it is of particular importance to estimate the correspondence of earlier and later life cognitive ability amongst premorbid samples. Poor correspondence would indicate that any discrepancy between the ‘baseline’ estimates derived at older ages and current ability should be interpreted with caution.

The present study examined repeated pre-adult IQ test scores of individuals during their school years as participants of a longitudinal study in Guatemala City, Guatemala and who were remeasured at ages 64–76 years. Repeated childhood and adolescent IQ scores, in combination with later life follow-up measures, are rare and come with high time and financial costs. Studies with individuals aged 60+ years are particularly rare, as the participants in most cohort studies have not yet reached this age. In this context, our analysis is novel and valuable.

Two predictions were tested: 1) IQ, as derived from age-appropriate standardized tests, remains relatively stable across childhood and adolescence–i.e., within subject variance and range are smaller than between subject variance and range; 2) older age cognitive function or IQ may be predicted from pre-adult IQ.

## Methods

### Participants

The study sample consisted of individuals aged 64 to 76 in the year 2017 (born 1941 to 1953) who attended the Colegio Americano de Guatemala (American School) and took part in Universidad del Valle de Guatemala’s (UVG) Longitudinal Study of Child and Adolescent Development [20]. The UVG longitudinal study ran between 1953 and 1999. A variety of measurements were taken annually, including age-appropriate standardized and validated mental ability/IQ tests. The original study investigated child physical and cognitive development in different socioeconomic status (SES) groups. The American School is an expensive private school and the students represent, generally, the highest SES group in the country [21]. All participants of the present study had access to private health care and good quality nutrition, making their general living conditions comparable to upper middle-class or high SES North Americans. We are focused on the American School students for the present study because it is the only school with participants who are currently aged 64+ years, and because it was deemed the best school to test the feasibility of following up on the UVG study participants, as the school has an active alumni network.

### Follow-up data collection procedure and sample representativeness

**Fig 1:**
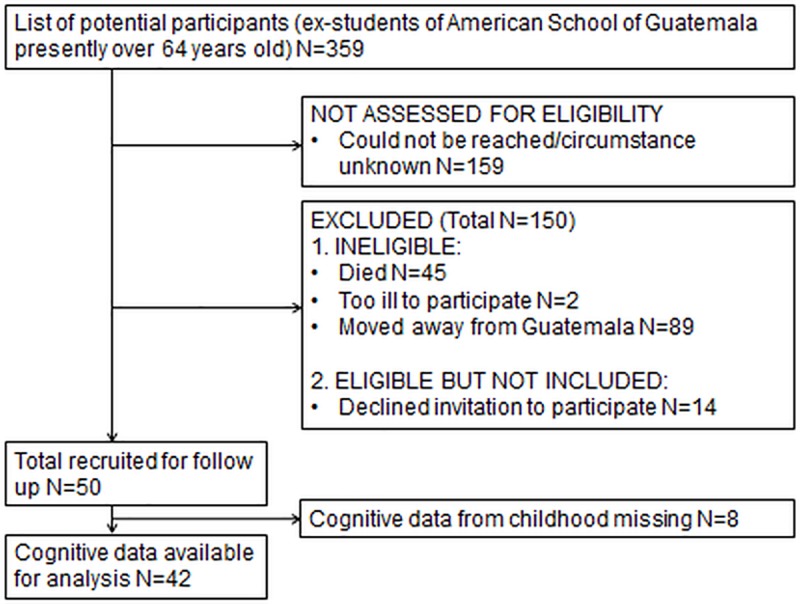
Flow chart of follow-up sample selection (N = 42).

We initiated a follow-up with participants from the UVG study in September 2016. The last contact with the current study participants was over 50 years ago. Participants were recruited using local contacts and alumni networks of the American School. We were able to include 42 participants of the original sample. This sample represents 14% of original study participants who met the selection criteria of: a) having preserved data on mental ability/IQ from at least one school year at the American School, and b) being currently aged 64 years or over. Many individuals could not be reached as no current contact information could be obtained, others had left the country and a considerable number had already died (Fig 1). The mean pre-adult IQ score for all individuals meeting the selection criteria was 105.60 (n = 359, SD = 11.95), which was 0.31 points lower than that in the follow-up sample. The age range of the individuals in the follow-up sample and the total pool of eligible participants was the same. There were 17 women and 25 men in the follow-up sample who had IQ measurements available of when they were between 5.21 and 17.13 years of age. Ethical approval was granted prior to onset of the study by the Ethics Approvals (Human Participants) Sub-Committee of Loughborough University REF NO: R16-P091 and the UVG Social Sciences Ethical Committee REF NO: 21, all participants signed an informed consent form in their native Spanish.

### Measures

Age-appropriate, group-administered, standardized and validated, Spanish-language paper and pencil mental ability/IQ tests were administered multiple times over the course of the study. Test administrators were given written instructions and received training. Testing time was 30 minutes for the total test, following the test instructions. The instruments used in the study were from the Otis Self-administering Tests of Mental Ability and the Pintner General Ability Test series. The instruments are listed in Table 1 along with information on sample sizes, mean scores, the school years and the student ages for administration of each test used for this sample. Despite some differences in the tests, all are scholastic aptitude and performance tests. These tests were developed in the early 20^th^ century. At time of testing, it was considered important to use age-appropriate tests to measure mental ability in children. Universal IQ tests were not suitable for the purpose of the study as these are time consuming and require specialist individual administration. The analysis of results from these tests was designed to calculate a standardized score, that is an IQ score with a mean of 100 and a standard deviation of 15. The IQ conversions from the raw test scores were done at time of testing by the UVG Study administrators. The raw scores were not archived, and the digitised dataset used in the present analysis only included the IQ scores. There is no information available on the estimated g-loadings of these tests, that is, of how these tests relate to generalized intelligence, sometimes called ‘g’.

**Table 1:** IQ tests used in the study.

Name of test	N participants	Mean score (IQ points unless otherwise stated)	Administered during	Mean age at testing (y)
Pintner Cunningham A^1^	22	102.2	Preschool, 1^st^ year of primary school	6.7
Pintner Durost A^1^	29	100.9	2^nd^ and 3^rd^ year of primary school	8.9
Otis Intermedio^1^	35	109.3	4^th^, 5^th^ and 6^th^ year of primary school, 1^st^ and 2^nd^ year of middle school	11.4
Pintner General Intermedio^1^	28	108.7	5^th^ and 6^th^ year of primary school	11.9
Otis Superior^1^	34	106.9	3^rd^ year of middle school, 1^st^ and 2^nd^ year of high school	15.4
Fluid intelligence test^2^	42	4.3 out of 13 points	Follow up study (64–76 years old)	69.2
Spanish WAT^3^	42	26.9 out of 30 points	Follow up study (64–76 years old)	69.2
WAT-Chicago^3^	42	37.3 out of 40 points	Follow up study (64–76 years old)	69.2

Names, sample sizes, means, school years, and ages of administration of each IQ test used for this sample. The protocol for the longitudinal study, particularly in the early years, saw changes from one year to the next.

^1^Translated to Spanish at Colegio Americano and Universidad del Valle de Guatemala with permission from copyright holders, copyright to Spanish versions held at Centro de Investigaciones Educativas (UVG)

^2^Lyall et al. (2016)

^3^Spanish Word Accentuation Test (WAT) Del Ser et al., 1997; 2015; Krueger, 2006

Detail on the precise cognitive abilities tested are not available for the tests, as some of the testing materials and related publications were not preserved after the end of the study in 1999. A partial scanned copy of instruction manual for the Pintner General Ability Tests exists, and identifies the following six testing categories: vocabulary, number sequence, analogies, opposites, logical selection and arithmetic reasoning [22]. These categories apply for the Pintner Cunningham A, Pintner-Durost A and Pintner General Intermedio tests–the main difference between these tests was in the age at which each was recommended for administration. IQ testing standards were less stringent in the past, and the booklet uses the terms ‘mental age’, ‘IQ’, ‘intellectual ability’, ‘reading ability’, and ‘general ability’ without precise definitions, and at times interchangeably. Overall, there is a clear emphasis on verbal-reasoning type tasks.

The Otis tests are similar to the Pintner tests, and also share a similarity with the verbal intelligence element of the Wechsler Adult Intelligence Scale. The Otis test sheets do not identify categories of abilities tested but include questions on vocabulary, arithmetic reasoning, synonyms and logical selection. There are partial available test sheets for most of the tests, and these show that all included highly similar elements, such as identifying the category to which a word belongs and understanding the meaning of proverbs. The original purpose of these tests, as of all early IQ tests, was to measure aptitude for scholastic performance. Previous work on longitudinal stability of IQ scores has made use of data from very similar tests to the current study. The Moray House Test No. 12 used in the Scottish Mental Health Survey, for example, also has an emphasis on verbal-reasoning type tasks and was used for several later-life follow-up investigations [11]. Most of the UVG Study data are well-preserved and were digitized at the Centro de Investigaciones Educativas UVG. There were some missing data in individual time series, particularly from the early years of the study, and the number of available pre-adult IQ test scores varied between individuals.

During the follow-up, new cognitive data scores were collected from 42 participants using the UK Biobank two-minute fluid intelligence test, the Spanish word accentuation test (WAT) and the Spanish word accentuation test Chicago version [23–25]. All these tests have been used to assess intelligence in middle-aged and older individuals [23–26], and were chosen as they are relatively rapid to administer. The UK Biobank fluid intelligence test includes 13 multiple choice questions assessing verbal and arithmetical deduction and has been validated in the UK for a middle-aged population [25]. No conversion table to IQ scores exists. The Spanish WAT and the Chicago WAT are essentially Spanish language versions of the National Adult Reading Test (NART), which has been extensively used to assess intelligence in older populations [27].There are high reported correlations between the results of the NART and universal intelligence tests [27]. The WAT tests have been used on smaller samples, and the authors of the Spanish WAT report a correlation of 0.84 between the test outcome and the Wechsler Adult Intelligence Scale [23]. All three tests in the follow-up were administered in the participant’s native language Spanish, and the fluid intelligence test was translated from English to Spanish by a bilingual specialist in cognitive testing and then back-translated and checked by an independent assessor. The Spanish language versions of the word accentuation tests have been validated, but the Spanish version of the UK Biobank two-minute fluid intelligence test has not been validated. In addition to the cognitive ability tests, the follow-up sample were screened for clinical signs of dementia using the official licenced Spanish version of the Mini Mental State Examination (MMSE) [28].

### Statistical analysis

Data were analysed with SPSS version 23 [29]. Descriptive analyses were used to explore variance in raw pre-adult IQ test scores between and within individuals. Spearman’s rank correlations were run between pre-adult test scores and age at time of testing to explore any consistent relationship between IQ score and participant age which may confound the results. Further Spearman’s rank correlations were run between pre-adult test scores and year of measurement to check for consistent bias in test administration year. A Tukey’s test was used to explore differences in means between the tests to investigate possible differences in test score distributions. The cognitive tests used for the follow-up provided raw scores and there are no IQ conversions available currently. To compare performance between the different tests we converted all scores to Z-scores using sample standard deviations.

To compare pre-adult scores with the later life tests, data needed to be reduced to one score per individual. Listed in Table 2 are the different rationales for choosing pre-adult test scores for their stability and predictive ability. A Spearman’s rank correlation matrix was used to assess which pre-adult IQ score was most suited for further analysis for this sample–the options identified in Table 2 were correlated against each other (first, last, maximum, and average of all available scores per individual, Table 3). These were further correlated against the three older age measures. Following selection of a representative childhood IQ (see Results), the difference between best representative pre-adult and the older age Z-scores was calculated and the individuals were categorised into Z-score gainers (increase of >1.0 SD), Z-score maintainers (within -1.0 to +1.0 SD) and Z-score losers (decrease of >1.0 SD).

**Table 2:** Overview of the different rationales for choosing pre-adult test scores for their stability and predictive ability.

Pre-adult standardized test score	Rationale for	Rationale against
First	No retesting effects.	Age at first test varies greatly due to missing data and students entering the school (and thus the study) at different time points.
Last	Maximum learning, presumably greatest cognitive capacity.	Age at last test varies greatly due to students leaving the school, missing testing events etc. There may not be a constant learning curve in the data
Maximum	Maximum achievement recorded for each student at any age; no missing data; least affected by poor motivation.	Needs to be controlled for age, difficult to interpret.
Average	Captures all information available for an individual.	Does not account for the variance in the range in scores between individuals, or in number of measures available per person.
Select one age	Age at testing automatically controlled for.	Many missing data; would result in a greatly reduced sample and will thus be excluded from Table 4.

**Table 3:** Correlations of pre-adult IQ tests scores.

	IQ test	Pintner Cunningham A	Pintner Durost A	Otis Intermedio	Pintner General Intermedio	Otis Superior
1	Pintner Cunningham A					
2	Pintner-Durost A	.012 (N = 13)				
3	Otis Intermedio	.128 (N = 13)	.232 (N = 16)			
4	Pintner General Intermedio	.232 (N = 11)	.248 (N = 20)	.265 (N = 12)		
5	Otis Superior	.204 (N = 14)	**.491** (N = 22)	.628* (N = 20)	.316 (N = 23)	

Spearman's rank correlation matrix of pre-adult IQ tests.

Bolding denotes statistical significance (p<0.05) and

* significance at p<0.01 level.

## Results

**Fig 2:**
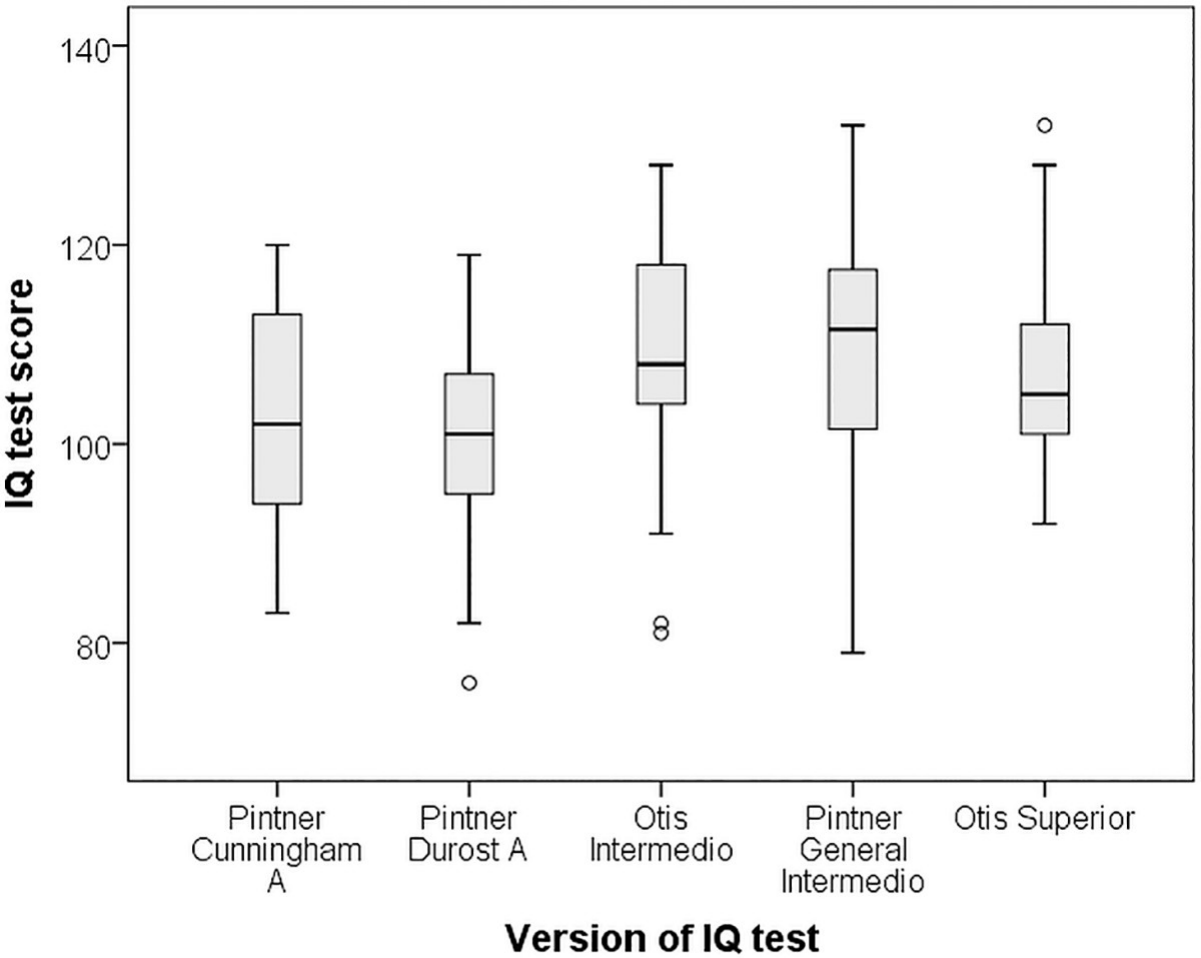
Pre-adult IQ test scores by test used. Box plot (median, quartiles, minimum, maximum) of IQ test scores by test used (Pinter Cunningham A (N = 22), Pinter Durost A(N = 29), Otis Intermedio (N = 35), Pinter General Intermedio (N = 28), and Otis Superior (N = 34).

Descriptive data on each childhood IQ test, mean scores and ages of participants are presented in Table 1. Neither age at testing nor year of measurement were significantly related to pre-adult IQ test scores (age: rho = 0.12, *p = 0*.*15*; for year of measurement: rho = 0.1, *p = 0*.*22*). There were some differences in score distributions between the tests used (Fig 2), and most between-test correlations were low (Table 3). The range in mean outcome scores between tests was up to 8.4 points. The Pintner Durost A test significantly differed in mean from the Otis Intermedio (mean difference = -8.45, *p = 0*.*025*). All other between test comparisons were non-significant.

The mean pre-adult IQ score, for all available datapoints, was 105.91 points (pre-adult IQ datapoints N = 153, SD = 11.18) and the range was 56 points (76–132). Within individuals, the mean range in pre-adult IQ scores was 15.19 points, ranging from 0 to 40 points. In 59.5% of participants, the fluctuation in scores was 15 or more points, and in 7.1% more than 30 points. The average standard deviation (SD) in pre-adult IQ scores within subjects was smaller than the SD of between subject IQ scores (SD = 7.0 vs. SD = 11.18). However, in 16.6% of participants the within subject SD in the scores was larger than the SD 11.18 points found in the group (SD ranging from 11.24 to 13.84 points). We reject prediction 1, that is, IQ, as derived from age-appropriate standardized tests, remains relatively stable across childhood and adolescence–i.e., within subject variance and range are smaller than between subject variance and range.

**Fig 3:**
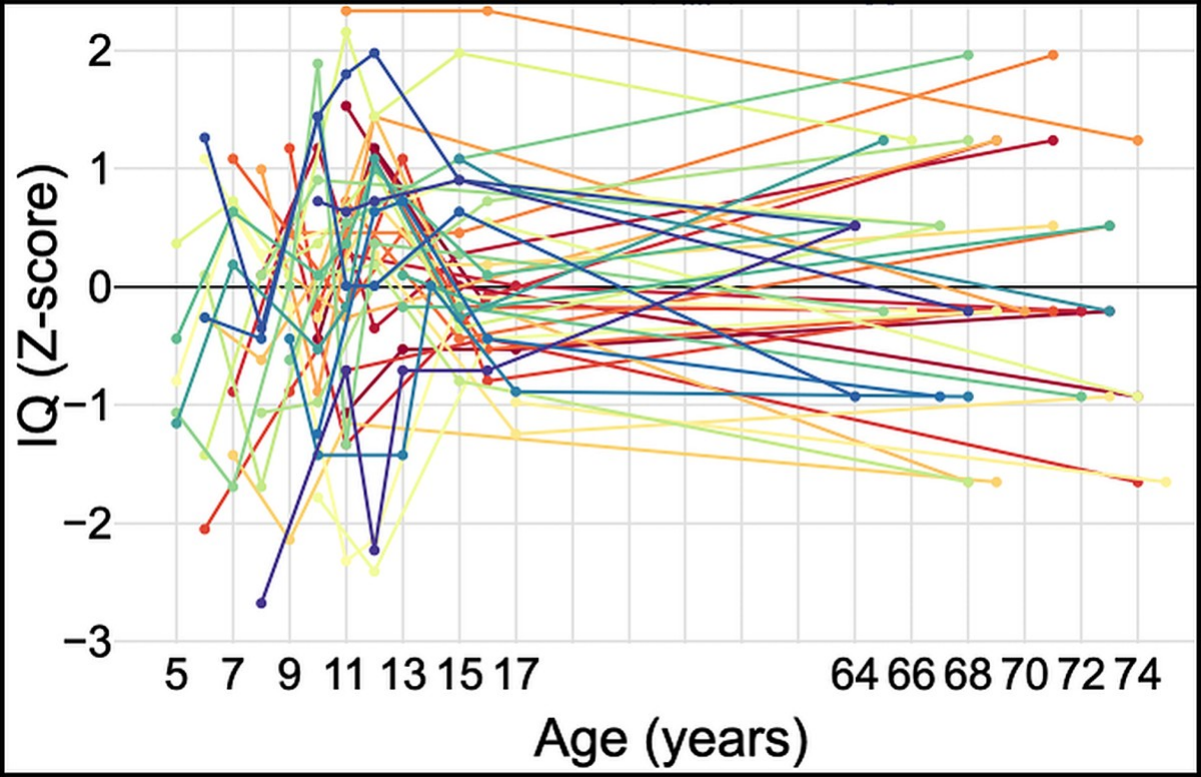
IQ Z-scores across the life course. IQ Z-scores by age, each line represents an individual. Data to age 17 years comes from the five cognitive tests administered as part of the UVG longitudinal study. Older age data come from the two-minute fluid intelligence test. Raw scores were converted to Z-scores using the sample standard deviations.

The correlation matrix comparing ways of selecting a representative pre-adult IQ score revealed much variation in the relationship each option had to the others, as well as with the later life outcome measures (Table 4; Fig 3). The follow-up participants did not show clinical signs of dementia based on the MMSE assessment: no participant scored below the clinical cut-off of 24 points, and the average score was 28.5 (SD 1.7, range 24–30). It was concluded analyses did not need adjustment for dementia status. The Spanish WAT Z-scores and the Chicago WAT Z-scores applied at ages 64+ correlated with each other but did not correlate significantly with average pre-adult cognitive test Z-scores or the fluid intelligence test Z-scores (Table 4). The average pre-adult Z-scores showed the highest correlations with other pre-adult scores and correlated significantly with the fluid intelligence Z-scores in later life (rho = 0.348, *p* = 0.024). We may interpret this to indicate that average IQ scores taken at different points over childhood are best in determining later life intelligence and that the fluid intelligence test was the most appropriate to asses later life IQ, but not reading tests. However, only a modest amount of total variation in the old age scores (12%) was explained by the pre-adult scores in this sample. When the pre-adult Z-scores were compared with the results of the fluid intelligence test Z-scores, 62% maintained their Z-score category (change in Z-score of less than 1), 19% saw a decrease (decrease in Z-score of more than 1) and 19% saw an increase (increase in Z-score of more than 1). On balance, we accept some evidence in favour of prediction 2, that is, subjects’ older age cognitive function is related to pre-adult IQ (measured as IQ Z-score), but only to a limited extent.

**Table 4:** Correlations of pre-adult IQ scores and later life test scores.

			Pre-adult				Later Life		
		Z-scores	First	Last	Maximum	Average	Fluid Intelligence	Spanish WAT	WAT Chicago
**Pre-adult**	1	First							
	2	Last	.493*						
	3	Maximum	**.440**	.643*					
	4	Average	.795*	.808*	.726*				
**Later Life**	5	Fluid intelligence	.089	.486*	.270	**.348**			
	6	Spanish WAT	.105	.127	.106	.161	.186		
	7	WAT-Chicago	-.106	.074	.037	.140	.140	.674*	

Spearman's rank correlation matrix of pre-adult IQ scores (N = 42) and later life cognitive test Z-scores (N = 42).

Bolding denotes statistical significance (p<0.05) and

* significance at p<0.01 level.

## Discussion

This study examined whether 1) IQ, as derived from age-appropriate standardized tests, remains relatively stable across childhood and adolescence–i.e., within subject variance and range are smaller than between subject variance and range; and 2) older age cognitive function or IQ may be predicted from pre-adult IQ. We found large intraindividual variability in IQ test scores in this sample of Guatemalan high SES individuals tested over their pre-adult years, and that the within individual distribution of IQ test scores varied greatly between participants. With regard to the second hypothesis, we found that the pre-adult IQ test scores were only modestly related to older age fluid intelligence test scores but not to WAT.

For longitudinal use of these data the results pose a challenge, as there are multiple ways in which the information collected over the participants’ growth period could be used in analyses. On balance, it was decided that the average pre-adult IQ test score best represented the individuals’ IQ before adulthood. The performance for the Spanish WAT and the Chicago WAT applied at ages 64+ were not related to either the pre-adult IQ or the fluid intelligence test scores. This suggests that the WAT tests are not necessarily measuring the same aspects of intelligence as the other tests, it could be that the WAT scores relate more to crystallised than fluid intelligence. There was also only a moderate correlation between average pre-adult IQ scores and the fluid intelligence test scores. The results imply that outcomes of one-off IQ tests taken before adulthood should be interpreted with caution, as individual variations in scores from year to year are likely to be high, at least in this study sample. As there has only been one follow-up testing event in old age, the results of the fluid intelligence test should be regarded with the same caution. There were no signs of clinical dementia in the sample, which leaves us to conclude that the discrepancy between childhood and old age test outcomes is not due to the influence of differential neurodegeneration rather than normal intraindividual variation in cognitive test performance.

The within individual longitudinal stability in pre-adult IQ test scores presented here is similar to values reported by previous, albeit scarce work on the topic. Fluctuations in general IQ in six to 18-year-olds have been reported to be more than 15 points in 58% of participants, and more than 30 points in 9% of participants in the Berkeley Guidance Study of 222 children born between 1928–1929 [30]. Similarly, Hutchens found that over 60% of her 113 participants had an IQ score fluctuation of at least one standard deviation in repeated testing events [13]. Both results are within three percent of the fluctuation found in the Guatemalan sample. Research reporting repeated cognitive test scores for children tends to cite between-test correlations to highlight good concurrence between the testing events [31–33]. In a Dutch twin study which used IQ test scores from ages 5, 7, 10 and 12 years, correlations of 0.64 to 0.78 between testing events were reported, but the study did not describe the range of variation found within individuals’ scores [31]. Schaefer and colleagues used an average IQ score in their study, but did not provide a rationale for their choice or any detail of the variation found in the scores of their sample [32].

Participants differed significantly in the progression of their IQ test scores. There were individuals in the group who very consistently achieved the same score year after year, whilst at the other end of the spectrum one individual’s fluctuation in scores was 40 points. In the Berkeley Guidance Study, it was reported that children whose mental test scores showed the most fluctuation often had life histories which showed unusual variations with respect to distressing factors, but there were other children whose scores remained constant despite upsetting experiences, such as conflict within the family [30]. As data on parental and other family relationships or traumatic events were not collected in the UVG Longitudinal Study, it is not feasible to do a similar comparison in the present study. The wider socio-political context in Guatemala may have played a role. The present sample grew up in Central America and had exposure to the Guatemalan civil war (1954–1996), violence, high crime rates, great political and social instability and very serious natural disasters during their youth [20,34,35]. Such events could have added variation to the test scores if a child was personally affected.

The IQ scores did not significantly correlate with age, implying there is no straightforward developmental cause behind the findings (the reference sample used to age-standardise the IQ test scores is not the present study sample, but that used by test manufacturers). The age-standardisations of IQ tests reflect a population average, and there may be individuals whose scores are related to a poorly matched age for developmental level. Many children mature earlier or later than the mean and thus at different stages of development may have a better or worse ‘fit’ with the test used in any given year. Mistakes may have been made in the data digitisation process as the results were copied from paper records. There was no significant correlation between the year the test was administered and the scores, implying that the results are not explained by any consistent error in test scoring between years. Several different tests were used from the Otis and the Pintner test series which may have added to the variation found.

The obvious limitation is the small study sample, which we hope to expand in the future. A larger sample might have shown greater consistency between measurements, but many studies with older participants will only be able to select sub-groups of the original sample with attrition due to mortality, moving away, unable to attend etc. This was the case with the present study as after 50 years recruitment to the follow-up study posed great challenges. The strength of the current study is the comprehensive assessment of responders and their repeated measures over childhood and later life.

The degree of stability and variability reported here between earlier vs. later life IQ scores fits well with existing evidence on life course trajectories of IQ. It is expected that there is stability in scores relative to peers through life, and we found that 62% of our sample had good tracking of IQ scores (within -1.0 to +1.0 Z-scores). It is not possible to pinpoint the factors which would lead a person to either a loss or gain in IQ scores, but it is possible that the 19% of individuals who performed worse in relation to the rest of the sample in old age compared to pre-adult may have experienced events over their life course which had a cognitive or physical toll. Factors related to cognitive performance in old age include social support and networks, good nutrition and physical and cognitive exercise, as well as risk and protective factors shared with cardiovascular disease [36–39]. These same factors may also explain why 19% increased their Z-score. Future research may investigate the extent to which these factors explain the variance in cognitive fluctuation over the lifespan.

Overall, the results presented here highlight the complicated nature of measuring and interpreting IQ at different ages, and the many factors that can introduce variation in the results. Large variation in the pre-adult test scores seems to be more of a norm than a one-off event. Over half of the participants of the present study showed variation of more than one standard deviation between testing sessions in pre-adult years. Averaging the scores before further analyses captures some of this fluctuation but it does not explain its existence in the first place. Is it good practice to use IQ scores from pre-adult years to predict and explain late-life health status? If there is only one score available, should it be used if we know that in the following year, the same child is quite likely to have a different result? This limitation should be highlighted in future studies using only one IQ assessment. On the other hand, the observed variation could yield very interesting lines of further research investigating what separates a child who scores consistently year after year from one who does not. Future research could also develop and evaluate psychometric instruments which better assess cognitive abilities and their variation when individuals are tested repeatedly during development.
